# Sustained free chlorine-releasing polydimethylsiloxane/Ca(ClO)_2_ materials with long-lasting disinfection efficacy

**DOI:** 10.1039/d4ra00663a

**Published:** 2024-04-16

**Authors:** Xiaofan Su, Yaqi Lin, Xingyu Hu, Xinzhi Tan, Yao Mai, Minyan Jiang, Ruitao Zhang, Fangjun Huo, Lei Liu, Weidong Tian, Li Xie

**Affiliations:** a State Key Laboratory of Oral Diseases & National Center for Stomatology & National Clinical Research Center for Oral Diseases & Engineering Research Center of Oral Translational Medicine, Ministry of Education & National Engineering Laboratory for Oral Regenerative Medicine, West China Hospital of Stomatology, Sichuan University Chengdu 610041 Sichuan China drtwd@sina.com samuel0121@163.com; b State Key Laboratory of Oral Diseases & National Center for Stomatology & National Clinical Research Center for Oral Diseases & Department of Oral and Maxillofacial Surgery, West China Hospital of Stomatology, Sichuan University Chengdu Sichuan 610041 China

## Abstract

A novel sustained chlorine-releasing polydimethylsiloxane/Ca(ClO)_2_ (PDMS/Ca(ClO)_2_) material was fabricated by encapsulating Ca(ClO)_2_ in a PDMS matrix due to its high hydrophobicity and high chemical stability, which showed immediate-responsive and long-lasting antibacterial capabilities in aqueous conditions. Free chlorine could be released from the PDMS/Ca(ClO)_2_ after immersion in water for 2 min and could also be sustainedly released for 2 weeks, while the released concentration is negatively related to the duration time and positively with the initial Ca(ClO)_2_ contents. Additionally, Ca(ClO)_2_ powder as a filler significantly affects the crosslinking and pore size of PDMS. The PDMS/Ca(ClO)_2_ materials exhibited enduring antibacterial performance against *Escherichia coli* (*E. coli*) and *Staphylococcus aureus* (*S. aureus*) in both planktonic and multispecies-biofilm status. It is expected that this PDMS/Ca(ClO)_2_ material and its similar composite would be promising candidates for wide sustainable disinfection applications in biomedical and industrial fields.

## Abstract 2

The article introduces a new PDMS/Ca(ClO)_2_ material that releases chlorine over two weeks, effectively combating bacterial growth, and suggesting versatile applications in biomedical and industrial disinfection.

## Graphical abstract

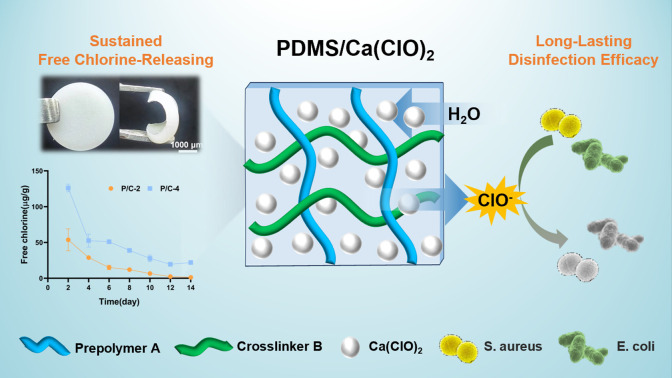

## 1. Introduction

Chlorine-based disinfectants are one of the earliest types of chemical disinfectants used by humans.^1^ Based on free chlorine, chlorine-based disinfectants possess strong oxidative properties. In light of their attributes encompassing broad-spectrum antimicrobial efficacy,^2,3^ cost-effectiveness,^4^ and user-friendliness,^2^ chlorine-based disinfectants showcase a wide range of applications, spanning from healthcare,^5^ environmental disinfection,^6,7^ and water purification^8,9^ to safeguarding food integrity^5,10–12^ and beyond.

Because harmful microorganisms are ubiquitous and have amazing reproductive capacity, which leads to the need for the use of disinfectants over and over again. Sustained-release type disinfectant agents could reduce the need for frequent administration, provide long-lasting antibacterial protection, save time and effort, avoid toxicity at high initial doses, and thus have great application potential in diverse fields. For example, by employing controlled-release mechanisms with minimal antibacterial agents, sustained-release antibacterial pads have achieved extended antibacterial effectiveness and prolonged the shelf life of cold fresh pork.^11^ The concentration of inorganic chlorine-based disinfectants (such as NaClO, Ca(ClO)_2_) in water drops rapidly,^13^ and in order to make the disinfectant work for a long time, Ca(ClO)_2_ tablets^14^ or polymers-carried chlorine disinfectants^15–19^ have been developed. For instance, Huang,^17^*et al.* developed a novel biobased chlorine-based sanitizer by encapsulating chlorine-binding polymer in a biobased yeast cell wall particle microcarrier, which could release free chlorine for only about 50 min. At present, some shortcomings including short sustained release time^17,18^ and complex preparation process^16^ have limited the widespread use and mass production of sustained-release chlorine-based disinfectants. It is important to explore novel strategies to enhance and prolong chlorine-based disinfectant release profiles for effective microbial control.

At present, a concerned method of sustained release of water-soluble drugs/reagents is mainly to encapsulate them in hydrophobic polymers. Among them, polydimethylsiloxane (PDMS) is a silicon-based polymer commonly used in numerous biomedical^20^ and industrial applications.^21,22^ Given to the presence of surface-oriented methyl groups from the dimethylsiloxane moiety, the surface of PDMS is highly hydrophobic but still allows slow water penetration.^23,24^ Therefore, encapsulating substances that are prone to react with water in a PDMS matrix can effectively slow down their reaction rate and prolong the reaction time. As a specific example, researchers have successfully developed a range of oxygen-producing materials by blending solid calcium peroxide with PDMS for tissue engineering studies.^25^ Additionally, PDMS is strictly chemically stable and does not react with chlorine oxidizers.^26^ Meanwhile, we chose Ca(ClO)_2_ as a chlorine source since Ca(ClO)_2_ boasts exceptional stability in its solid state, facilitating convenient storage and transportation. Free chlorine is supposed to be leisurely produced as a result of the Ca(ClO)_2_–H_2_O reaction.^27^

Therefore, we introduced a novel PDMS/Ca(ClO)_2_ composite material as a sustained-release chlorine disinfectant product. Firstly, the physicochemical properties of the synthesized materials were characterized by scanning electron microscopy (SEM), wettability assay, and so on. Subsequently, the release performance of Ca(ClO)_2_ from PDMS was systematically evaluated by *N*,*N*-diethyl-*p*-paraphenylenediamine (DPD) method, and Inductively Coupled Plasma Optical Emission (ICP-OES) Spectromete for a continued 2 weeks. Furthermore, we specifically investigated the long-lasting antibacterial activity of the PDMS/Ca(ClO)_2_ system against common pathogenic bacteria in both planktonic and biofilm forms.

## 2. Materials and methods

### 2.1 Preparation of PDMS/Ca(ClO)_2_

The PDMS (SYLGARD™ 184 silicone elastomer kit, Dow Corning) is a two components system consisting of prepolymer A (contains: dimethyl siloxane, dimethylvinylsiloxy-terminated; dimethylvinylated and trimethylated silica; ethylbenzenne) and crosslinker B (contains: siloxanes and silicones, dimethyl, methyl hydrogen; dimethyl siloxane, dimethylsioxy-terminated; dimethylvinylated and trimethylated silica). A vinyl-terminated PDMS with a platinum (Pt)-based catalyst and methyl hydrogen siloxane are the main functional components of prepolymer A and crosslinker B, respectively. The prepolymer A and the crosslinker B was mixed at a 10 : 1 weight ratio. And then different contents (0%, 2%, 4%, 6% w/v) of Ca(ClO)_2_ powders (available chlorine content: 28–32%, Fuchen Chemical, China) were added and poured into different molds (24-well tissue culture plates with a diameter of *Φ*15.6 mm per well, 12-well plates tissue culture plates with a diameter of *Φ*22.1 mm per well, 6-well plates tissue culture plates with a diameter of *Φ*34.8 mm per well) to prepare materials with the same volume (400 μL per well) but different surface areas. Then PDMS/Ca(ClO)_2_ mixture was vacuumed to remove air bubbles and cured at 50 °C for 3 hours. PDMS/Ca(ClO)_2_ samples with different contents of Ca(ClO)_2_ powders (0%, 2%, 4%, 6% w/v) were referred to as PDMS, P/C-2, P/C-4, P/C-6 respectively.

### 2.2 Physicochemical properties of PDMS/Ca(ClO)_2_

#### 2.2.1 Rheological properties

The rheological properties of the samples were determined using a rheometer (HAAKE^©^ Viscotester iQ). The storage modulus (*G*′) and loss modulus (*G*′′) were measured at shear rates ranging from 0.01 Hz to 10 Hz at a temperature of 25 ± 2 °C.

#### 2.2.2 Scanning electron microscope (SEM) and energy-dispersive X-ray (EDX) mapping

A scanning electron microscope (SEM, Inspect F, FEI, and Thermo Fisher Apreo 2C, USA) was used to observe the surface morphology of different groups. The spatial distribution of powder particles within the PDMS matrix after curing has been characterized by EDX mapping. All the samples were gold sputtered and then the analyzing procedures were carried out.

#### 2.2.3 Wettability assay

Drop Shape Analyzer (DSA30, KRUSS, German) was employed to measure the static contact angles and determine the surface wettability by the sessile drop method. To ensure reliability, we conducted a minimum of three measurements at different locations on both the PDMS and PDMS/Ca(ClO)_2_ surfaces with varying concentrations of Ca(ClO)_2_. Water, in the form of 2 μL drops, was utilized as the liquid medium in all of these contact angle experiments.

#### 2.2.4 Brunauer, Emmett and Teller (BET)

The N_2_ adsorption/desorption isotherms were obtained using a Micromeritics Tristar 3000 adsorption analyzer at liquid nitrogen temperature under a continuous adsorption condition. Prior to testing, the samples were degassed under a vacuum at 120 °C for at least 6 hours. The specific surface area of the samples was calculated using the Brunauer, Emmett, and Teller (BET) method based on the adsorption data, and the average pore size of PDMS/Ca(ClO)_2_ samples was calculated using the Barrett–Joyner–Halenda (BJH) model based on the adsorption branch of the isotherm.

### 2.3 Measurement of free chlorine and Ca^2+^ generation from PDMS/Ca(ClO)_2_

To assess the sustained generation of free chlorine, the primary component of chlorine-based disinfectants, from PDMS/Ca(ClO)_2_ materials, an *N*,*N*-diethyl-*p*-paraphenylenediamine (DPD, Aladdin, China) assay was conducted, which is the most common analytical method for the determination of free chlorine in water.^28^ Briefly, PDMS/Ca(ClO)_2_ materials prepared in different molds with varying Ca(ClO)_2_ contents (2% and 4% w/v) were soaked in ddH_2_O (1 mL, RT) separately while being protected from light. Firstly, the short-term release performance of the PDMS/Ca(ClO)_2_ samples prepared in 24-well tissue culture plates was evaluated by testing the free chlorine within 20 min. Long-term (14 days) release of free chlorine from the PDMS/Ca(ClO)_2_ samples prepared in different molds was also evaluated. In detail, every two days, the soaking solutions from each group were collected completely. The collected solutions were then diluted with ultrapure water, and 50 μL of DPD solution (10 mM) was mixed with 1 mL of diluted solution from each sample. Subsequently, the optical density (OD) value of the mixture was measured at 324 nm to evaluate the free chlorine sustained generation of the PDMS/Ca(ClO)_2_ samples. The coloration or tint of the samples may provide insights into the free chlorine sustained generation in the PDMS/Ca(ClO)_2_ samples.

The Ca^2+^ concentrations in the soaking solutions were tested by ICP-OES (Inductively Coupled Plasma Optical Emission Spectrometer, PE Avio 200, USA). Simply put, the experimental procedure involves placing P/C-2 and P/C-4 samples prepared in 24-well tissue culture plates in individual containers containing 1 mL of ddH_2_O. These containers will be kept in a light-shielded environment at room temperature. At regular intervals of two days, the soaking solution will be collected from each container, and a fresh 1 mL of ddH_2_O will be added for further soaking.

### 2.4 The antibacterial activity in planktonic forms

*Staphylococcus aureus* (*S. aureus*) and *Escherichia coli* (*E. coli*) were cultured separately in brain-heart perfusion (BHI) liquid medium at 37 °C with shaking at 200 rpm overnight. Prior to use, the bacteria were centrifuged and diluted to 10^5^ CFU mL^−1^. To evaluate the antimicrobial properties of the material, PDMS/Ca(ClO)_2_ materials prepared in 24-well tissue culture plates were used in this part. And four groups were set as follows: (1) control group (400 μL 0.9% saline), (2) PDMS, (3) P/C-2, (4) P/C-4. Then, 1 mL of bacterial suspension is co-cultured with the PDMS/Ca(ClO)_2_ samples from each group and soaked on different days (0, 4, 7, 14 days)in 12-well plates. After incubation for 12 h at 37 °C, the culture solutions were transferred into sterilized tubes and diluted 10^5^ times with PBS solution, and 20 μL of the bacterial suspension was coated onto 1/3 Soybean Casein Digest Agar Plate (TSA plate, HuanKai Microbial Co., Ltd, China) TSA plates. Subsequently, the plates were incubated for another 24 h at 37 °C before counting the bacteria CFU and taking photographs. S0, S4, S7, and S14 indicate that the samples were soaked in ddH_2_O for 0, 4, 7, and 14 days.

In order to observe the morphological alteration of bacteria, the bacteria after co-culturing with PDMS/Ca(ClO)_2_ samples for 12 h were fixed for 2 h at RT with 2.5% glutaraldehyde. The samples were then dehydrated using a gradient of ethanol solutions and finally observed by SEM.

The bacteriostasis rate was determined from the following formula: antibacterial rate (%) = (1 − colony forming units (CFUs) after treatment/CFUs before treatment) × 100%.

### 2.5 The antibacterial activity in biofilm forms

To evaluate the antibacterial activity in biofilm forms, *E. coli* and *S. aureus* were co-cultured on a titanium disc to obtain a dual-species biofilm. Briefly, *E. coli* and *S. aureus* were diluted separately with Brain Heart Infusion Broth (BHI Broth, Solarbio, China) to a concentration of 10^5^ CFU mL^−1^. Subsequently, 500 μL of each bacterial suspension was inoculated onto the surface of titanium discs and allowed to form biofilms at 37 °C for 48 hours. In order to improve the biofilm's adhesion, 1% (w/v) sucrose was added to the bacterial medium. Meanwhile, PDMS/Ca(ClO)_2_ samples prepared in 24-well tissue culture plates which were soaked in ddH_2_O (1 mL) for 7 days were dried at real temperature. The biofilm-coated titanium discs were then co-cultured in PBS with each group of PDMS/Ca(ClO)_2_ samples prepared, respectively, for 12 hours at 37 °C. After treatment, the titanium discs were rinsed with saline three times. Live and dead bacteria were stained with a LIVE/DEAD™ BacLight™ Bacterial Viability Kit (Thermo, USA) and visualized using confocal laser scanning microscopy (OLYMPUS FV1200, Japan). The antibacterial rates were calculated using Image J.

### 2.6 Statistical analysis

All data obtained were described as mean ± standard deviation (SD). With the help of GraphPad Prism version 8.0.2 for Windows, one-way ANOVA was used for statistical analysis. The probability value (*p*-value) < 0.05 was considered statistically significant. Statistical significance: *p < 0.05, **p < 0.01, ***p < 0.001. Unless otherwise specified, there are three parallel samples in each experimental group.

## 3. Results and discussion

### 3.1 Fabrication and characterization of the PDMS/Ca(ClO)_2_ materials

**Fig. 1:**
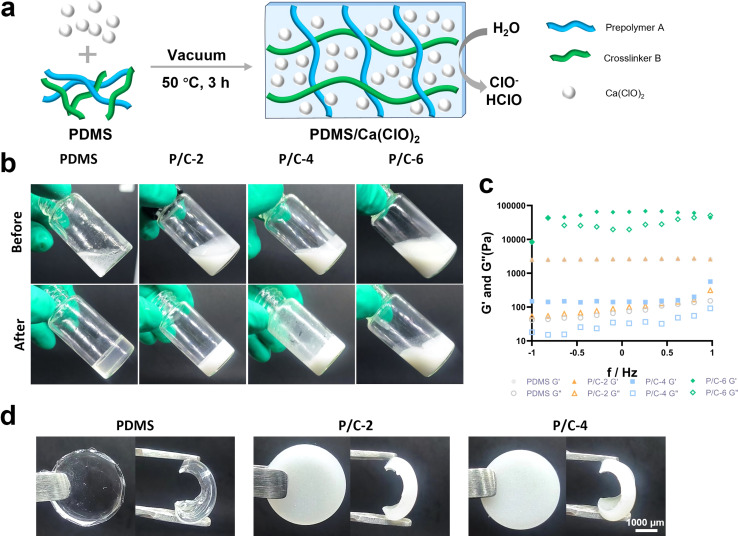
Preparation and characterization of PDMS/Ca(ClO)_2_ materials. Schematic illustration of the preparation of the materials (a). Photographs (b) and rheological properties (c) of PDMS/Ca(ClO)_2_ samples in glass bottles with different concentrations of Ca(ClO)_2_ before and after cross-linking. Photographs of PDMS/Ca(ClO)_2_ samples (d).

The synthetic route for the fabrication of the PDMS/Ca(ClO)_2_ samples was illustrated in Fig. 1a. The PDMS prepolymer A was mixed with Ca(ClO)_2_ powder at different concentrations (0%, 2%, 4%, and 6% w/v) along with the crosslinker B, and the mixture was vacuumed and cured at 50 °C for 3 hours. As shown in Fig. 1b, PDMS, P/C-2, and P/C-4 changed from a fluid to a solid state, indicating a complete crosslinking. In contrast, it can be observed that the P/C-6 sample did not fully solidify, suggesting that the excessive addition of Ca(ClO)_2_ powder affected the crosslinking of PDMS. This may be due to the strong alkalinity of Ca(ClO)_2_, which might disrupt the Si–H bonds of crosslinker B and prevent subsequent hydrosilylation crosslinking reactions between Si–H bonds (crosslinker B) and vinyl groups of siloxane (prepolymer A),^29^ though further verification of this hypothesis is required.

Furthermore, the rheological properties of the samples were determined using a rheometer. The storage modulus (*G*′) and loss modulus (*G*′′) are presented in Fig. 1c. Compared with the PDMS sample, the P/C-2 sample showed no significant change and the P/C-4 sample exhibited a lower *G*′. Moreover, The *G*′ and *G*′′ values of the P/C-6 sample showed that *G*′ was lower than *G*′′ with increasing shear rate, also indicating the material exhibited liquid-like characteristics.^30^ Therefore, in the subsequent experiments, only P/C-2 and P/C-4 samples were chosen.

The PDMS sample displayed a typical colorless and transparent appearance. After adding Ca(ClO)_2_, the P/C-2 and P/C-4 samples showed a milky white color and a decrease in transparency as the content of Ca(ClO)_2_ increased (Fig. 1d). Additionally, from the graph, it is observed that Ca(ClO)_2_ powders are generally uniformly distributed within the PDMS matrix.

**Fig. 2:**
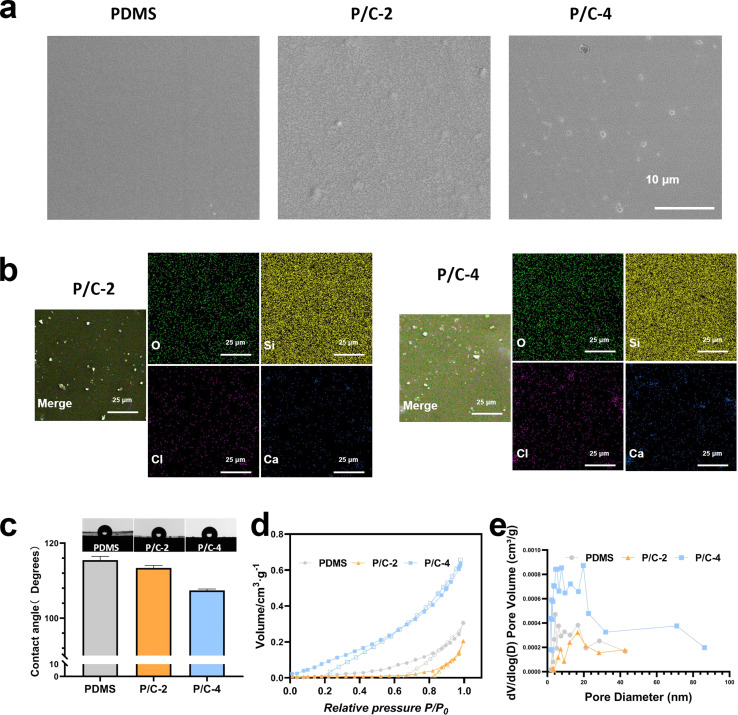
Physicochemical properties of PDMS/Ca(ClO)_2_. SEM images of PDMS/Ca(ClO)_2_ samples (a). EDX mapping of samples (b). Contact angles of samples (c). The N_2_ adsorption–desorption curves (d) and pore size distributions by BJH method(e).

The SEM results (Fig. 2a) revealed that the surface of the PDMS sample is exceptionally smooth and compact. On the surfaces of the P/C-2 and P/C-4 samples, the presence of Ca(ClO)_2_ particles can be observed.

The spatial distribution of powder particles within the PDMS matrix after curing has been characterized by EDX mapping (Fig. 2b). From Fig. 2b, it can be observed that both Ca and Cl are evenly distributed in the PDMS matrix, and in P/C-4, the Cl and Ca are significantly higher than in P/C-2.

A water contact angle test was employed to assess the wettability of the samples. The results in Fig. 2c revealed that all the samples were hydrophobic. However, compared with the PDMS group, the contact angles of P/C-2 and P/C-4 samples were slightly reduced. This could be probably attributed to the hydrophilic and dissolvable Ca(ClO)_2_ particles exposed on the PDMS surface. Next, as depicted in Fig. 2d, analysis of the N_2_ adsorption–desorption data revealed that the PDMS/Ca(ClO)_2_ samples exhibit a well-structured mesoporous morphology. The average pore size of PDMS, P/C-2, and P/C-4 samples (Fig. 2e) was calculated to be about 8.3 nm, 6.4 nm, and 14.1 nm, respectively. These findings suggest that the incorporation of 2% Ca(ClO)_2_ into the PDMS matrix has decreased the pore size of PDMS and 4% incorporation caused an increase. This may be due to the powder acting as a filler, filling the pores of PDMS and thereby reducing porosity when incorporating 2% Ca(ClO)_2_ into the PDMS matrix. However, at 4% incorporation, the normal cross-linking of the PDMS matrix may be affected due to two reasons. One aspect involves the physical space obstruction caused by powders,^31^ while the other aspect pertains to the disruption of Si–H bonds in the crosslinker B due to the strong alkalinity of Ca(ClO)_2_,^29^ leading to an unexpected increase in porosity.

### 3.2 Monitoring of free chlorine release, pH values and Ca^2+^ release

The hydrolysis of Ca(ClO)_2_ primarily yields ClO^−^ and Ca^2+^. The reaction primarily involves two processes:

1 Ca(ClO)_2(s)_ + 2H_2_O_(l)_ → Ca(OH)_2(s)_ + 2ClO^−^_(aq)_ + 2H^+^_(aq)_

2 Ca(OH)_2(s)_ + 2H_2_O_(l)_ → Ca^2+^_(aq)_ + 2OH^−^_(aq)_

**Fig. 3:**
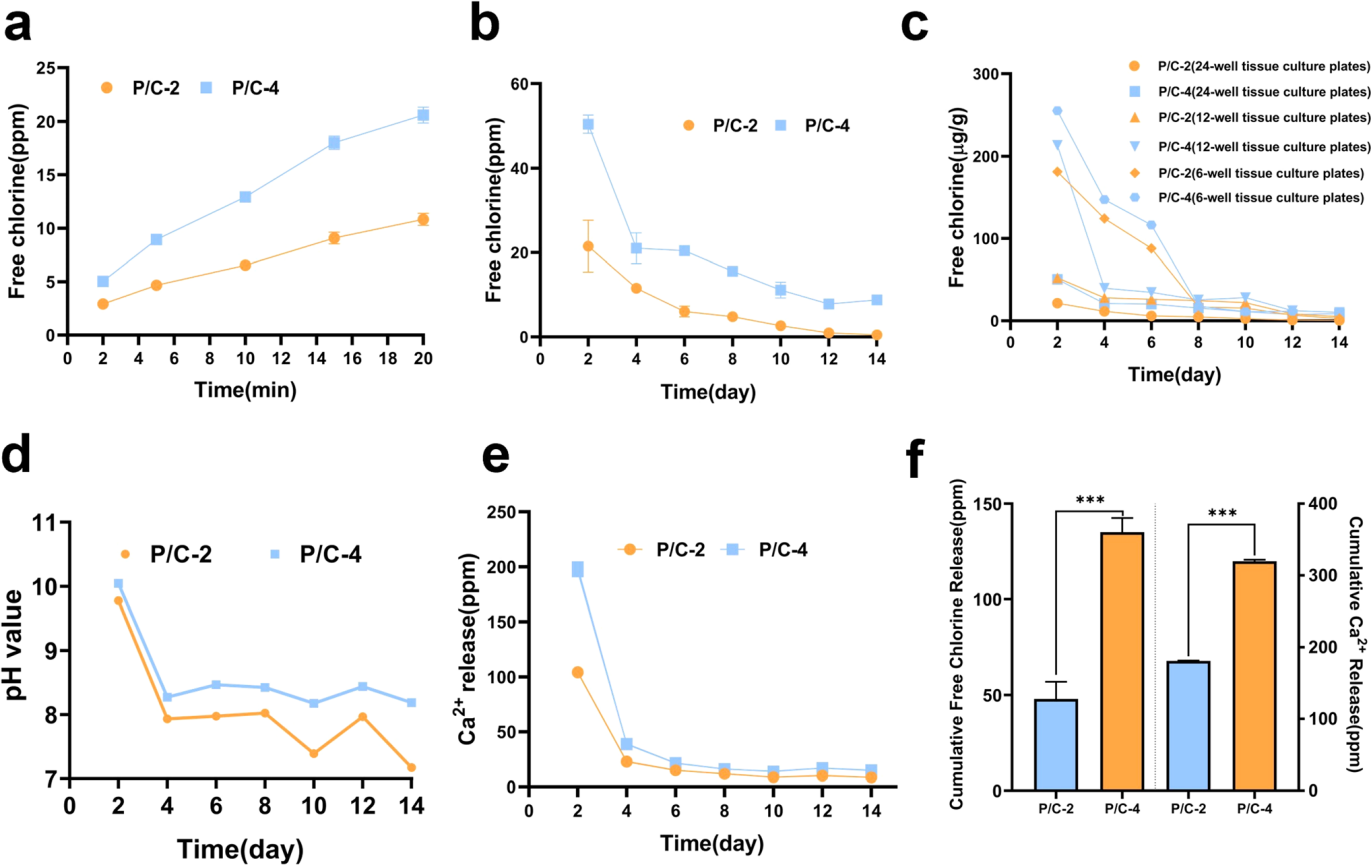
Assessment of sustained release capability. Release curves of free chlorine from the discs prepared in 24-well tissue culture plates (*Φ*15.6 mm per well) within 20 minutes (a) and over 14 days (b). Release curves of free chlorine from the discs prepared in 24-well tissue culture plates (*Φ*15.6 mm per well), 12-well plates tissue culture (*Φ*22.1 mm per well), and 6-well plates tissue culture plates (*Φ*34.8 mm per well) over 14 days (c). The pH variation curves in the soaking liquids from the discs prepared in 24-well tissue culture plates (d). Ca^2+^ release curves from the discs prepared in 24-well tissue culture plates (e). The cumulative Ca^2+^ and free chlorine release amount over 14 days (f). (ns indicates no significance, *p < 0.05, **p < 0.01, ***p < 0.001).

The release of free chlorine from PDMS/Ca(ClO)_2_ samples in short and long duration was detected using the DPD method. The results (Fig. 3a) showed that free chlorine can be released quickly within 2 min from both PDMS/Ca(ClO)_2_ groups prepared in 24-well tissue culture plates. In addition, the cumulative release amount of free chlorine increased over time during 20 min' monitoring. The P/C-4 sample released more free chlorine than P/C-2, which was consistent with the amount of added Ca(ClO)_2_.

Fig. 3b showed that free chlorine could be sustainedly released for about 14 days for both of the two groups from 24-well tissue culture plates and the P/C-4 group released more and seemed to last longer. Moreover, it can be noted that the average released concentration was positively related to initial Ca(ClO)_2_ contents and also gradually decreased with time. The cumulative release amount of free chlorine after 14 days (Fig. 3f) depicted that the total release concentration for the P/C-4 sample was about three-fold higher than that of P/C-2. It is notable for proposing that the high chemical stability of PDMS also avoids the assumption of free chlorine by the PDMS matrix. Meanwhile, release curves of free chlorine from the discs prepared in different molds (Fig. 3c) showed that under the premise of identical PDMS volume and Ca(ClO)_2_ content, a larger surface area corresponds to a faster and more abundant release of free chlorine within a 14 days period. Hence, we can tailor materials with diverse Ca(ClO)_2_ concentrations and surface areas to meet specific application demands.

The pH values of the soaking solution (ddH_2_O) from materials prepared in 24-well tissue culture plates for 2–14 days are shown in Fig. 3d. In the P/C-2 and P/C-4 groups, although the pH initially exceeded 10 on day 2, it maintained a weak alkaline condition with pH values between 7–8.5 across the subsequent test period. The relatively mild alkalinity is environment-friendly and beneficial for safe application. According to literature,^32^ the ratio of different types of free chlorine species including ClO^−^, HClO, and Cl_2_ is pH-dependent. Under pH 10, ClO^−^ is the most predominant, while at pH values 7–8.5, a small portion of HClO exists. It is also reported that HClO possesses stronger antimicrobial activity compared with ClO^−^.^33^

The release of Ca^2+^ from PDMS/Ca(ClO)_2_ samples was detected by ICP-OES. As shown in Fig. 3e, the amounts of Ca^2+^ were about 200 mg L^−1^ and 100 mg L^−1^ on day 2 for P/C-4 and P/C-2 samples, respectively. Then, the concentration sharply decreased to about 40 ppm on day 4 and remained at ∼10 ppm till day 14. This further confirmed that Ca(ClO)_2_ in PDMS/Ca(ClO)_2_ samples reacted with water and released the reaction products.

### 3.3 The long-term antibacterial activity in planktonic forms

**Fig. 4:**
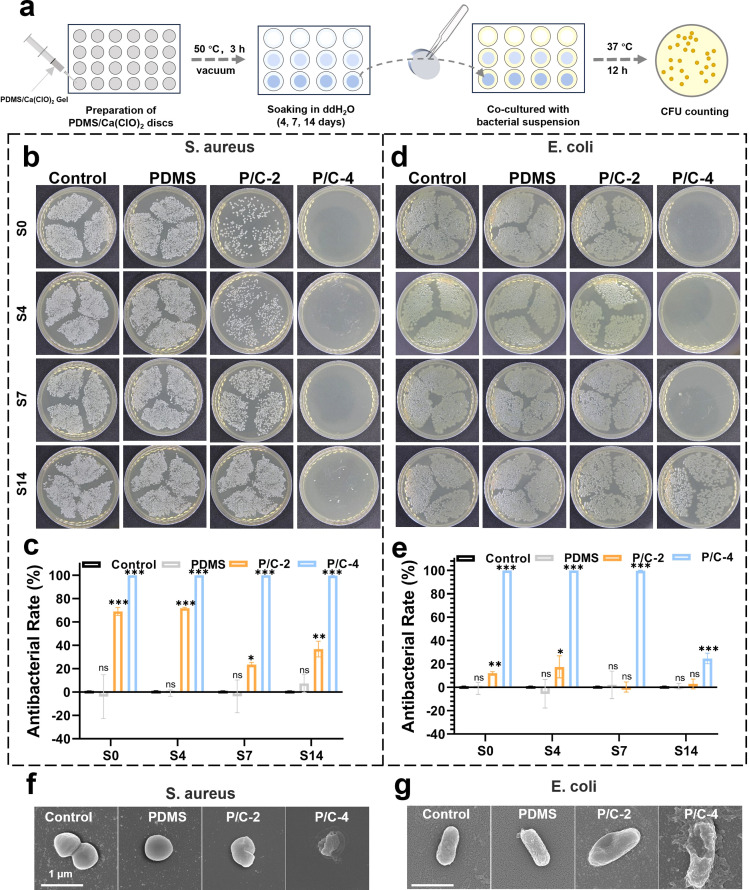
Long-term antibacterial activity in planktonic forms. Schematic diagram of antibacterial experiments in planktonic forms (a). Photographs of bacteria colonies cultured of *S. aureus* (b) and *E. coli* (d). S0, S4, S7, and S14 indicate that the samples were soaked in ddH_2_O for 0, 4, 7, and 14 days. Percentage of survivals of *S. aureus* (c) and *E. coli* (e). SEM images of *S. aureus* (f) and *E. coli* (g) after being treated for 12 h with different samples. (ns indicates no significance, *p < 0.05, **p < 0.01, ***p < 0.001).

To investigate the long-term disinfection activity of PDMS/Ca(ClO)_2_ in planktonic forms, *S. aureus* and *E. coli* were selected as test organisms. The antibacterial efficacy of the PDMS/Ca(ClO)_2_ samples was evaluated using the CFU counting method. As shown in Fig. 4a, the bacterial suspension was co-cultured with PDMS/Ca(ClO)_2_ samples, which were soaked in 1 mL ddH_2_O for 0, 4, 7, and 14 days in order to mimic the different service durations. As shown in Fig. 4b, the antibacterial rates of P/C-2S0 and P/C-2S4 samples were about 70% against *S. aureus* and those of the P/C-2S7 and P/C-2S14 samples were about 20–40%. The P/C-4 samples exhibited a stronger antibacterial effect against *S. aureus* than P/C-2. The P/C-4S0, P/C-4S4, and P/C-4S7 samples exhibited antibacterial rates close to 100%. Even after 14 days of soaking in water, the antibacterial rate of the P/C-4S14 group was still ∼99.68%. In contrast, the PDMS samples had no antibacterial effect, similar to the control group.

The antibacterial data of *E. coli* are displayed in Fig. 4d and e. Generally, the antibacterial rates of the samples against *E. coli* are lower compared with *S. aureus*. The antibacterial rates of P/C-2S0 and P/C-2S4 samples were about 20% against *S. aureus* and those of the P/C-2S7 and P/C-2S14 samples had weak antibacterial ability. Notably, the P/C-4S0, P/C-4S4, and P/C-4S7 samples exhibited antibacterial rates close to 99.7%. Even for the P/C-4S14 samples, it exhibited an antibacterial rate of ∼24.7%. This difference is probably attributed to the surface charge and structure of the bacteria. Gram-negative bacteria typically carry a negative charge on their surface, while Gram-positive bacteria, like *S. aureus*, carry a positive charge. In an alkaline environment, free chlorine exists mainly as negatively charged ClO^−^, favoring interactions with the negatively charged surface of Gram-negative bacteria. Besides, the thinner cell walls of Gram-positive bacteria, are more susceptible to oxidation and damage.^34^ In contrast, Gram-negative bacteria possess more complex cell walls with a lipid bilayer and lipopolysaccharide layer, providing antioxidative and exclusion properties that render them less susceptible.^35,36^ SEM images (Fig. 4f and g) showed the morphology alteration of the bacteria after different treatments, which were generally consistent with the above CFU results. In comparison to the control and PDMS groups, *S. aureus* treated with P/C-4 exhibited noticeable rough shrinkage in morphology, while the bacterial morphology of the P/C-2 group showed slight deformation and roughness. The overall trend of morphological changes in *E. coli* was similar to that of *S. aureus*, but it showed minor cell rupture.

### 3.4 The long-term disinfection activity in biofilm forms

**Fig. 5:**
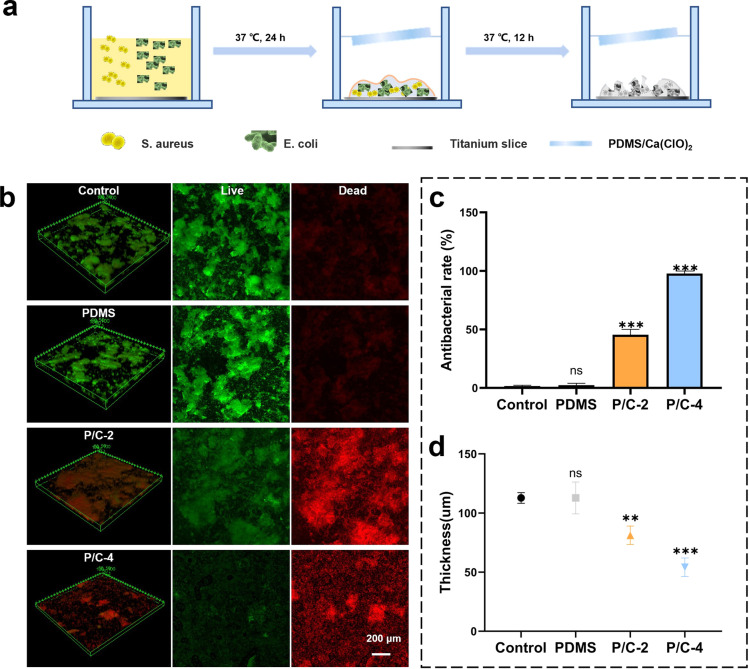
Long-term antibacterial activity in biofilm forms. Schematic illustrations of preparation of dual-species biofilm and experiments of antibacterial in biofilm forms (a). Live/dead staining confocal laser scanning images of dual-species bacterial biofilms after treatment with different samples (b). Antibacterial rates statistical chart corresponding to bacterial biofilms treated with different samples (c). Biofilm thickness statistical chart corresponding to different material treatments (d). (ns indicates no significance, *p < 0.05, **p < 0.01, ***p < 0.001).

The antibacterial activity against biofilms formed on titanium discs was further evaluated using Live/dead staining and CLSM observation. The schematic illustration of the preparation of dual-species biofilm (*E. coli* and *S. aureus*) and experimental design is shown in Fig. 5a. In order to exhibit the long-term effects, all the used samples were soaked in ddH_2_O for 7 days and dried before follow-up antibiofilm experiments. The trends observed were consistent with the antibacterial activity data obtained from planktonic cultures (Fig. 3). As shown in Fig. 5b and c, the P/C-4S7 group exhibited the highest antibacterial ratio against the mixed biofilm (∼97.8%), followed by the P/C-2S7 group with an antibacterial ratio of ∼45.5%. The pure PDMS samples showed no antibacterial effect. Furthermore, upon analyzing the biofilm thickness (Fig. 5d), it was observed that the P/C-2 and P/C-4 samples exhibited significantly thinner biofilm compared to the PDMS samples, suggesting that the samples could also eliminate biofilm extracellular polymeric substances (EPS) in some extent.

The study showcases a meaningful achievement, as the utilization of PDMS/Ca(ClO)_2_ composite materials allows for the sustained release of free chlorine for long-term disinfection or antibacterial applications. Free chlorine can be rapidly released after being immersed in water, also satisfying immediate-response requirements. The antibacterial results robustly established its enduring antimicrobial capabilities to both Gram-positive and Gram-negative bacteria. It is worth pointing out that the preparation procedure of PDMS/Ca(ClO)_2_ materials is rather simple and environmentally friendly, beneficial for industrial production and wide use. Besides, due to the excellent processability of PDMS, this material can be processed into various shapes, such as membranes, spheres, sheets, *etc.*, to meet different application requirements. Nonetheless, in the future, it is essential to address the issue that higher Ca(ClO)_2_ contents lead to the uncrosslinking of PDMS, so as to enhance the flexibility and concentration-adjustability of the system. We firmly assert that the PDMS/Ca(ClO)_2_ materials have great promise in diverse applications, such as but not limited to disinfection for cistern water^37^ and dental unit waterlines.^38^ Additionally, this work proposed a novel sustained-release disinfectant design by utilizing the unique silicone rubber-based polymer PDMS, which might shed light on wider research and product development potentials for diverse disinfectants and silicone materials.

## 4. Conclusion

In this study, we have demonstrated the generation of free chlorine-releasing composite materials through a simple combination of PDMS and Ca(ClO)_2_. The Ca(ClO)_2_ incorporated within the PDMS matrix exhibits sustained and slow reactions with water in aqueous environments, resulting in a continuous release of free chlorine for 2 weeks. Moreover, the PDMS/Ca(ClO)_2_ composite retained effective long-term antibacterial activity in both planktonic and biofilm forms. Considering these promising results, we believe that PDMS/Ca(ClO)_2_ material holds great potential for various applications such as water treatment and medical fields as a novel sustained-release disinfectant.

## Author contributions

Xiaofan Su: conceptualization, methodology design, data collection, data analysis, data interpretation, writing – original draft. Yaqi Lin: methodology design, data collection, data analysis, visualization. Xingyu Hu: methodology design, writing – review & editing. Xinzhi Tan: data collection, writing – review & editing. Yao Mai: experimental design, data analysis, visualization. Minyan Jiang: methodology design, writing – review & editing. Ruitao Zhang: data analysis. Fangjun Huo: data analysis, methodology design. Lei Liu: project management, supervision. Weidong Tian: project leadership, conceptualization, fundraising. Li Xie: corresponding author, conceptualization, fundraising, writing – review & editing.

## Conflicts of interest

The authors declare no competing financial interest.

## Supplementary Material
